# Profile and antimicrobial susceptibility patterns of bacteria isolated from effluents of Kolladiba and Debark hospitals

**DOI:** 10.1515/biol-2022-0960

**Published:** 2024-09-11

**Authors:** Tamene Milkessa Jiru, Ewunetu Ayanaw

**Affiliations:** Department of Biotechnology, Institute of Biotechnology, University of Gondar, P.O. Box: 196, Gondar, Ethiopia; Department of Environmental and Industrial Biotechnology, Institute of Biotechnology, University of Gondar, P.O. Box: 196, Gondar, Ethiopia

**Keywords:** antimicrobial susceptibility, bacterial profile, effluents, plasmid profile

## Abstract

This study aimed to investigate the presence of antibiotic susceptibility patterns and bacterial profiles of some multi-drug-resistant bacteria isolated from the effluents of Kolladiba and Debark Hospitals. Sixteen samples were collected from Kolladiba and Debark Hospitals in North Gondar, Ethiopia, to investigate the presence of multi-drug-resistant bacteria. To assess susceptibility patterns, well-isolated bacterial colonies were subjected to seven antibiotics. The selected resistant isolates were characterized using morphological and biochemical tests. Plasmid DNA analysis of the isolates was also performed. Out of a total of 28 bacterial isolates, 12 were found to be multi-drug resistant. Among the tested antibiotics, erythromycin was the most resistant antibiotic, while novobiocin was the most effective antibiotic. A plasmid profile study of the isolates revealed both the presence and absence of plasmids. The number of plasmids ranged from zero to four, with plasmid sizes of 100, 900, 1,000, 1,400, 1,500, and 1,800 base pairs. This study concluded that effluents from both hospitals have high number of multi-drug-resistant isolates. The genes responsible for multi-drug resistance in bacterial isolates under this study could be either plasmid-mediated or chromosomal DNA-mediated. The presence of multi-drug-resistant bacteria in these effluents should not be overlooked.

## 1 Introduction

Serious infections caused by pathogenic bacteria that have become resistant to commonly used broad-spectrum antibiotics have become major global healthcare issues in the current century, and also, the growth of antibiotic-resistant bacteria in hospital setups is becoming a serious problem. Such emerging antibiotic resistance is threatening the management and control of bacterial infections. Although antibiotic resistance as an environmental problem is increasing from time to time, it has largely been overlooked, and prevention and containment have received far too little attention. As a result, the increasing incidence of resistance to a wide range of antibiotics by a variety of pathogenic organisms is a major concern facing modern medicine. Furthermore, antibiotic resistance has resulted in increased healthcare costs, morbidity, and mortality among patients with infectious diseases [1].

In Africa, including Ethiopia, many hospitals and clinics are major sources/reservoirs for large numbers of disease-causing bacteria, and such bacterial strains are introduced into the public environment [2]. The spread of antibiotic-resistant bacteria from hospitals to the environment can occur through various mechanisms, such as discharged antibiotics that are excreted through feces and urine of patients, discharged patients and health-care workers, accidental spilling of different antibiotics, and discarding expired drugs without burning. Overuse and misuse of antibiotics have accelerated antibiotic resistance. Uncontrolled and unrestricted uses of different antibiotics by the community also cause a rise in antibiotic resistance and result in the spread of resistance genes in hospital effluents. These multiple antibiotic-resistant pathogenic bacteria entering into an environment mainly through effluents are causing a serious public health impact. Bacteria resistant to these antibiotics, which carry the transmissible gene, have the ability to transfer such transmissible genes to other normal non-resistant bacterial communities. Such infections caused by the transferred resistance gene of bacteria are habitually difficult to treat [3]. It also minimizes the power of the antibiotic pool for handling bacterial infections [3]. Hence, hospital effluents could raise a number of common antibiotic-resistant bacteria in the recipient environment both by the mechanisms of introduction and selection pressure [4]. It is also true that hospitals provide an environment conducive to multi-drug bacteria, making the treatment options limited and expensive [5].

Genes coding for antibiotic resistance and virulence may share common features of being located in the bacterial chromosome, as well as on plasmids. The genes they carry may be expressed under stressed conditions and are considered to be living organisms in their simple structure since they are the main elements of a continuous lineage with every individual’s evolutionary history [6]. Many pathogenic bacteria have plasmids that carry antibiotic resistance encoding genes, which contribute to the emergence of multi-drug resistance [7,8]. These plasmids also encode virulence factors.

In the study areas, there is a lack of information on whether hospital effluents carrying antibiotics could contribute to the development of antibiotic-resistant microorganisms in the environment or not. The current study was designed to assess the antimicrobial susceptibility pattern of bacteria isolated from the effluents of two hospitals in North Gondar, Amhara Region, Ethiopia, against commonly used antibiotics. The study also aimed to analyze the plasmid profile of the multi-drug-resistant isolates. The purpose of studying the plasmid profile was to check whether the resistance is plasmid-mediated or chromosome-mediated. The findings of this study therefore would help to make public health authorities aware of the dissemination of drug-resistant bacteria in the hospital environments for proper management of effluents.

## 2 Materials and methods

### 2.1 Description of the study areas

**Figure 1:**
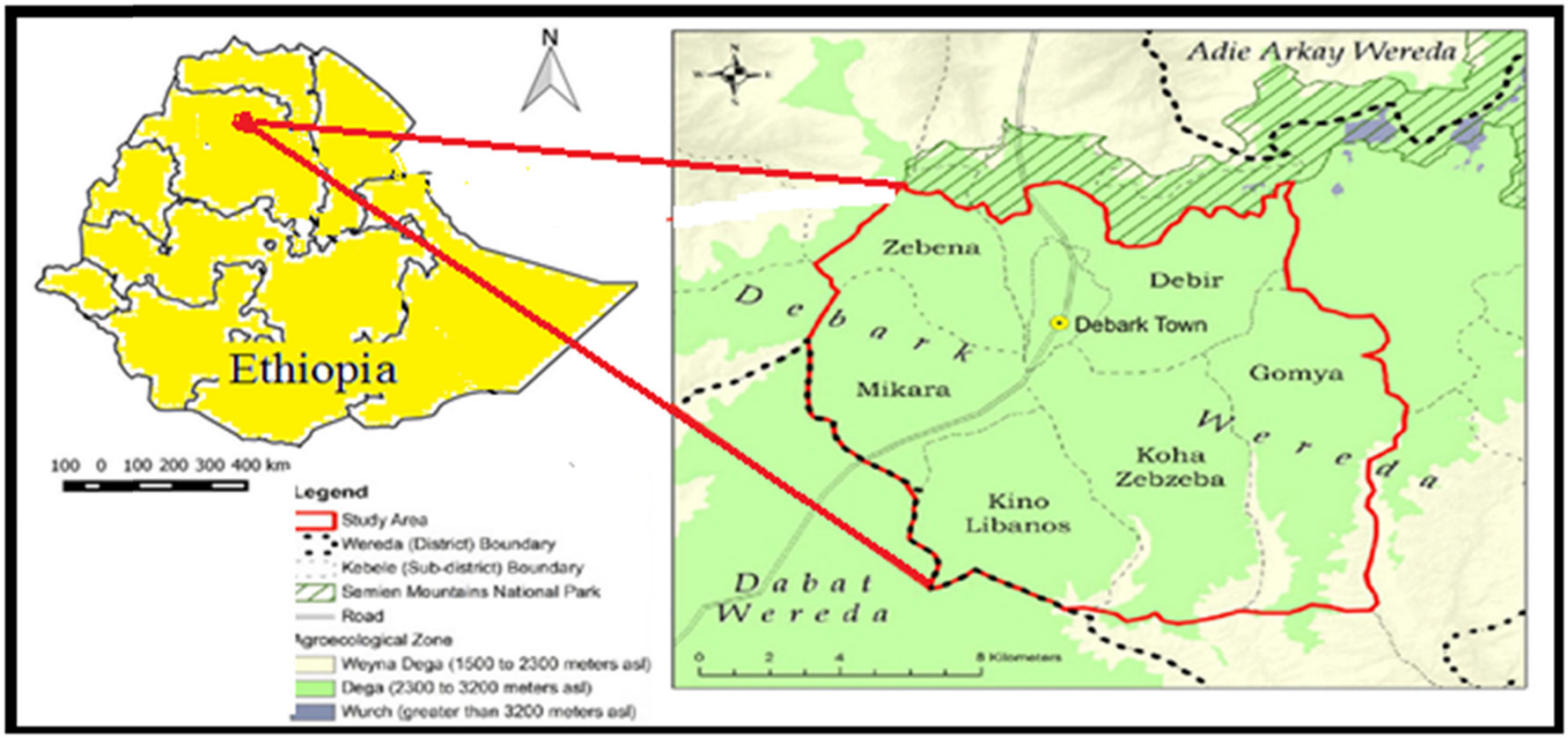
Map showing study area – Debark district (Debark town) [9].

**Figure 2:**
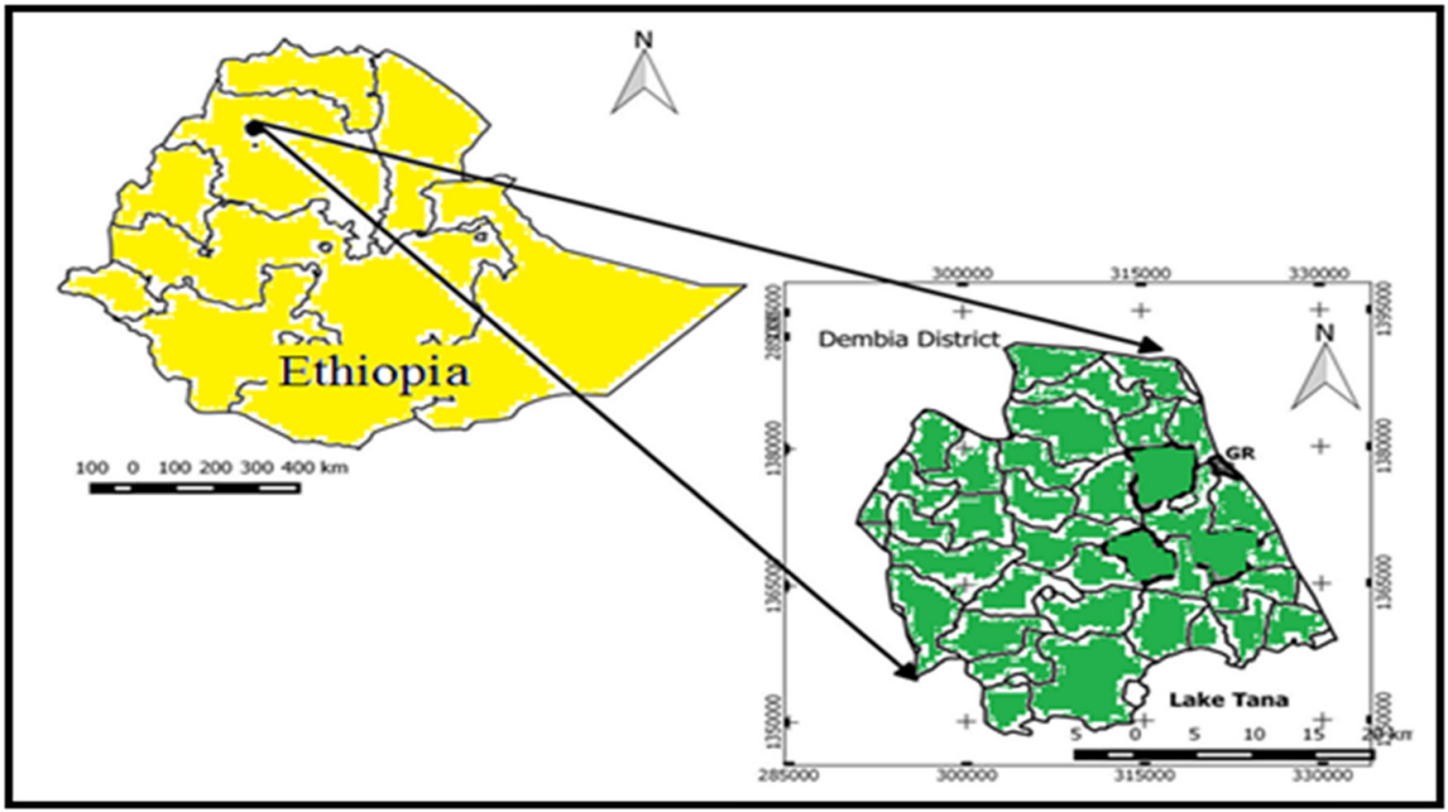
Map showing Dembia district (Kolladiba town).

The study was carried out in Debark and Kolladiba hospitals of North Gondar, Amhara Region, Ethiopia. Debark is located in the northern part of Gondar town near Ras Dashen mountain. The town has 13°08′N latitude and 37°54′E longitude with an elevation of 2,850 m above sea level. Conversely, Kolladiba is 16 km far from Gondar town in the southwestern part. Kolladiba town has 12°40′N latitude and 37°10′E longitude with an elevation of 2,146 m above sea level. The distances from Addis Ababa to Kolladiba and Debark are 729 and 835.3 km, respectively. Maps of both study areas are depicted in Figures 1 and 2.

### 2.2 Sample collection and isolation of bacteria

A cross-sectional study was used to conduct this research. A total of 16 effluent samples were collected from Debark and Kolladiba hospitals in North Gondar, Amhara Region, Ethiopia. They were taken from different sites of the two hospitals (around the drug store, laboratory, shower, surgical room, and patient bedding room) and finally at the sites where all the hospital effluents collectively enter into the septic tank. The samples were collected using sterilized plastic bags. The collected samples were transported to the Microbial Laboratory, Department of Biotechnology, Institute of Biotechnology, University of Gondar. Then, the samples were preserved at 4°C in a refrigerator for further analysis.

Bacteria were isolated by a serial dilution method. Stock solution was prepared by diluting 1 mL of the effluent sample in 9 mL of distilled water and shaken using a vortex mixer. From the stock solution, 1 mL was used to prepare the final volume of 10^−4^, 10^−5^, 10^−6^, and 10^−7^ and distributed on Mueller Hinton agar medium aseptically. Then, the plates were incubated at 37°C for 48 h. Distinct bacterial colonies that were grown well on each plate were observed. Distinct representative colonies were sub-cultured on differential and selective media such as mannitol salt agar (*Staphylococcus* sp. confirmation test), MacConkey agar (*Escherichia coli* isolation test), Salmonela–Shigella agar (*Salmonella* and *Shigella* confirmation test), pseudomonas agar (*Pseudomonas* sp. confirmation test), and XLD (xylose lysine deoxycholate) agar media (*Salmonella* confirmation test) [10].

### 2.3 Morphological and biochemical characterization tests

The selected isolates underwent morphological and biochemical analysis [11].

### 2.4 Morphological tests

The morphology of growing bacterial colonies, such as size, color, shape, margin, elevation, texture, appearance, and opacity of each isolate, was tested and used for identification purposes [11].

### 2.5 Biochemical characterization

Isolates were characterized using various biochemical tests following the procedures set in Bergey’s Manual of Systematic Bacteriology and Determinative Bacteriology [11], including Gram straining, motility and catalase tests, citrate utilization tests, coagulase and urease production tests, indole production tests, oxidase tests, and Methyl Red–Voges Proskauer (MR–VP) tests.

### 2.6 Antibiotic susceptibility test

The antimicrobial susceptibility profile of isolates was assessed using the standard Kirby Bauer disk diffusion assay [12]. The inoculum of each bacterium was prepared by suspending the freshly grown bacteria in 5 mL sterile nutrient broth, and the turbidity was adjusted to an approximate value of 0.5 McFarland standard. Then, the antibiotic discs were placed over Mueller Hinton agar plates seeded with each bacterial isolate. The common antibiotics used for susceptibility tests were ampicillin (AMP, 10 µg), tetracycline (TET, 30 µg), gentamycin (GEN, 10 µg), amoxicillin (AMX, 10 µg), novobiocin (NV, 30 µg), erythromycin (E, 15 µg), and cotrimoxazole (COT, 25 µg). The Petri plates were incubated at 37°C for 24 h. The zone of growth inhibition around each disc was measured, and the results were interpreted according to the guidelines from the Clinical and Laboratory Standards Institute [13]. All experiments were done in triplicates. The experimental results were expressed as the mean of three replicates.

### 2.7 Plasmid DNA isolation and analysis

Those selected antibiotic-resistant isolates were subjected to plasmid analysis [14]. The isolates were grown in 10 mL of nutrient broth in test tubes and incubated at 37°C for 18–24 h. After incubation, 1,500 µL of each culture was transferred into 2 mL Eppendorf tubes for plasmid extraction, and glycerol was added to the remaining culture and stored at 4°C. The 1,500 µL bacterial culture in the Eppendorf tubes was centrifuged at 6,000 rpm for 8–10 min and placed in ice. Then, the supernatant was carefully removed using a fine-tip micropipette, and the pellet was thoroughly re-suspended in 100 µL of ice-cold solution I (containing sucrose and Tris–ethylene diamine tetra acetic acid). The suspension was vortexed gently, placed in ice for 5 min, and then shifted to room temperature.

After that, 200 µL of solution II (containing sodium hydroxide and sodium dodecyl sulfate) was added to the suspension containing pellets and solution I. Those pellet–suspension mixtures were mixed by inverting the mixture six to seven times, and the tube was maintained for 1 min at 0°C.

Then, 150 µL of solution III (containing acetic acid and potassium acetate) was added from the ice box to the sample containing test tubes. The content of each tube was gently mixed by inverting the tubes for five to six times and maintained at 0°C for 5 min. The tubes were centrifuged for 10 min at 6,000–8,000 rpm to yield a clear supernatant. This fractionation step separates the plasmid DNA from the cellular debris and chromosomal DNA in the pellet. Plasmid DNA renatures and remains in solution. Other components get precipitated. The supernatant was removed carefully without disturbing the pellet from each tube and transferred into new Eppendorf tubes, and 200 µL of ethanol was added to it. The precipitate was collected using centrifugation at 10,000 rpm for 20 min. Then, the supernatant was decanted, and the Eppendorf tube was inverted on bloating paper to drain left over, which was still stuck to the Eppendorf tube at the bottom side. The inverted tube was dried for about 10–15 min at room temperature. The dried pellet was suspended with 50 µL of 1× TE buffer and mixed by tapping by finger; the extracted plasmid DNA was quantified using nanodrop, and then the plasmid DNA in solution was applied to an agarose gel for electrophoresis. The samples were processed using horizontal gel electrophoresis to identify the number of plasmid copies present in different isolates of each cell. For this purpose, an agarose gel of 1% was used. Staining of DNA fragments was carried out using ethidium bromide (0.5 μg/mL), and they were visualized by UV-Trans illumination equipment. A standard DNA molecular weight marker was used to estimate the plasmid size. The standard DNA marker was used in this 1 kb ladder with 100 bp ladder gaps. This was done to check whether the isolated bacterial-resistant gene is plasmid-mediated or chromosomal DNA-mediated.

## 3 Results and discussion

### 3.1 Bacterial colony isolation and subculture and screening

In this study, a total of 16 effluent samples were collected from Kolladiba and Debark hospitals. Of these samples, 28 bacterial isolates were recovered. The bacterial isolates from Debark hospital effluent samples were designated as SD1, SD2, SD3, SD4, SD5, SD6, SD7, SD8, SD9, SD10, SD11, SD12, SD13, SD14, SD15, and SD16, while that of Kolladiba effluent samples were labeled as SK1, SK2, SK3, SK4, SK5, SK6, SK7, SK8, SK9, SK10, SK11, and SK12. The isolates were sub-cultured using differential and selective media. From these bacterial isolates, 12 (SD1, SD2, SD4, SD7, SD11, SD15, SD16, SK2, SK3, SK5, SK7, and SK8) were found to be multi-drug resistant. By the way, multi-drug resistance is defined as non-susceptibility to at least one agent in three or more classes of antibiotics.

### 3.2 Morphological and biochemical characteristics of multi-drug resistant bacteria

Colony morphology is the visual culture characteristics of a bacterial colony on an agar media. Observing colony morphology is an important skill used in the microbiology laboratory for the identification of microorganisms. Colonies need to be well isolated from other colonies to observe the characteristic shape, size, color, surface appearance, and texture. The different colony morphology characteristics, i.e., colony shape, size, texture, appearance, etc., were characterized for each of the 12 bacterial isolates. The details are described in Table 1.

**Table 1:** Colony characteristics of selected bacterial isolate

Isolate	Colony characteristics							
	Size	Color	Shape	Margin	Elevation	Texture	Appearance	Opacity
SD1	1.0 mm	Bright yellow	Circular	Entire	Convex	Mucoid	Rough	Opaque
SD2	2 mm	Grey	Circular	Entire	Convex	Shiny	Smooth	Opaque
SD4	2.2 mm	White	Irregular	Entire	Raised	Shiny	Smooth	Transparent
SD7	1.5 mm	Yellow	Filamentous	Entire	Raised	Shiny	Smooth	Opaque
SD11	2 mm	Pale yellow	Circular	Entire	Convex	Shiny	Smooth	Transparent
SD15	1.8 mm	Colorless	Circular	Undulate	Raised	Shiny	Smooth	Opaque
SD16	1.9 mm	Yellow	Filamentous	Entire	Convex	Mucoid	Smooth	Transparent
SK2	0.8 m	Yellow	Spindle	Entire	Convex	Mucoid	Smooth	Transparent
SK3	2.6 mm	Colorless	Circular	Undulate	Raised	Shiny	Smooth	Opaque
SK5	1 mm	Golden yellow	Circular	Entire	Convex	Mucoid	Rough	Opaque
SK7	2.5 mm	White	Circular	Entire	Convex	Mucoid	Smooth	Opaque
SK8	2.0 m	White	Circular	Entire	Raised	Shiny	Smooth	Transparent

In this study, ten biochemical tests were performed against each isolated multi-drug-resistant bacteria from effluent samples. Variations were observed for the different tests across the isolates. Each of the biochemical test results is presented in Table 2.

**Table 2:** Biochemical test results for bacterial isolates from effluent Debark and Kolladiba hospital sample

Tests	SD1	SD2	SD4	SD7	SD11	SD15	SD16	SK2	SK3	SK5	SK7	SK8
Gram staining	+	−	−	+	−	−	−	−	−	+	−	−
Shape	Spherical	Rod	Rod	Spherical	Rod	Rod	Rod	Rod	Rod	Spherical	Rod	Rod
Indole test	−	−	+	−	−	−	−	−	−	−	−	+
Catalase test	+	+	+	+	+	+	+	+	+	+	+	+
Oxidase test	+	+	−	+	−	+	+	+	+	+	−	−
Urease test	−	−	−	−	−	+	−	−	+	−	−	−
Coagulase test	+	−	−	+	−	−	−	−	−	+	−	−
Citrate test	+	−	−	+	−	−	+	+	−	+	−	−
Motility test	−	+	+	−	−	+	−	−	+	−	+	+
Methyl red	+	−	+	+	+	−	−	−	−	+	+	+
VP test	+	−	−	−	−	−	+	+	−	+	−	−
Isolate name	*Staphylococcus* sp.	*Citrobacter* sp.	*E. coli*	*Staphylococcus* sp.	*Shigella* sp.	*P. aeruginasa*	*K. pneumoniae*	*K. pneumoniae*	*P. aeruginosa*	*Staphylococcus* sp.	*Salmonella* sp.	*E. coli*

In this study, different bacterial colonies were isolated from hospital effluents and identified using morphological characteristics (shape, size, color, texture, margin, and elevation of the colony) and different biochemical tests (Gram staining, urease, catalase, motility, oxidase, indole production, coagulase, citrate, and MR-VP tests) [11]. Based on the morphological and biochemical test results, isolates were assigned as *Staphylococcus* sp. (SD1, SD7, and SK5), *Citrobacter* sp. (SD2), *E. coli* (SD4 and SK8), *Shigella* sp. (SD11), *P. aeruginosa* (SD15 and SK3), *K. pneumoniae* (SD16 and SK2), and *Salmonella* sp. (SK7).

### 3.3 Antibiotic susceptibility tests

The 28 bacterial isolates that were recovered from effluent samples were subjected to antibiotic susceptibility tests using seven commonly used antibiotics. Almost all of the commonly used antibiotics that were tested in this study failed to inhibit all 12 bacterial isolates, while the rest isolates were sensitive to all 7 antibiotics (Table 3).

**Table 3:** Resistance rate of bacterial isolates from Debark and Kolladiba Hospital effluent samples against commonly used antibiotics

Bacterial isolates	Antibiotic resistance *N* (%)						
	AMP	AMX	COT	GEN	NV	TET	E
*Staphylococcus* sp. (*n* = 3)	3(100)	2(66.7)	3(100)	3(100)	3(0)	2(66.7)	3(100)
*E. coli* (*n* = 2)	1(50)	1(50)	1(100)	1(50)	1(50)	2(100)	2(100)
*P. aeruginosa* (*n* = 2)	2(100)	2(100)	1(50)	1(50)	1(50)	2(100)	2(100)
*Salmonella* sp. (*n* = 1)	1(0)	1(0)	1(0)	1(100	1(0)	1(100)	1(100)
*Citrobacter* sp. (*n* = 1)	1(0)	1(100)	1(100)	1(0)	1(100)	1(0)	1(100)
*Shigella* sp. (*n* = 1)	1(100)	1(100)	1(100)	1(100)	1(0)	1(0)	1(100)
*K. pneumoniae* (*n* = 2)	2(100)	2(100)	2(0)	2(100)	2(100)	2(100)	2(100)

AMP: Ampicillin, TET: Tetracycline, GEN: Gentamycin, AMX: Amoxicillin, NV: Novobiocin, E: Erythromycin, COT: Cotrimoxazole.

Susceptibility to antibiotics result presented a varying degree of resistance to the different antibiotics tested against the tested bacteria. However, all isolates were sensitive to at least one of the seven antibiotics except against erythromycin (100% resistance). One isolate of *E. coli* (SK8) was 100% resistant to all the tested antibiotics. When we take into consideration about isolates showing resistant to more than five antibiotics, the distribution of multi-drug-resistant isolates in Debark and Kolladiba hospitals is still found to be high.

Antibiotic resistance is a major public health threat, and the presence of resistant organisms in environmental wastewaters/effluents is an emerging concern around the world. In this study, high incidences of antibiotics-resistant bacteria were isolated from Kolladiba and Debark Hospitals effluents. Out of 28 isolates, 12 were found to be resistant to at least one to seven tested antibiotics. This appeared to be analogous to what was predicted in many reports by researchers in Ethiopia that there is clear evidence of abuse of antibiotics for which the emergence of multi-drug-resistant bacteria is continuously increasing from time to time [15,16]. The indiscriminate use of antibiotics (misuse and overuse of antibiotics) currently has resulted in the increase of occurrence of antibiotic-resistant bacteria in the hospital effluents. The presence of a high rate of multi-drug-resistant bacteria in the hospital effluent is an alarm to take measures. This is because it may further result in disease spread and treatment failure. Similar results have been reported by Praveenkumarreddy et al. [17] in India and Molla et al. [18] in Gondar, Ethiopia. In this study, the antimicrobial susceptibility pattern shows that the isolates were highly resistant to the most commonly used antibiotics. One isolate of *E. coli* (SK8), which was isolated from the Kolladiba Hospital effluent sample, was 100% resistant to all the tested antibiotics (Table 3). Conversely, there was a high degree of resistance to erythromycin (100% resistance). The most effective antibiotic was novobiocin, with 58.3% inhibition effectiveness against the tested bacteria. A similar result was reported by Teshome et al. [19] in eastern Ethiopia, who isolated samples from hospital effluents. Study results by Osadebe and Okounim [20] also demonstrated that waste effluent from hospital environments contains a wide range of multi-drug-resistant bacteria.

### 3.4 Plasmid isolation and bacterial profile

**Figure 3:**
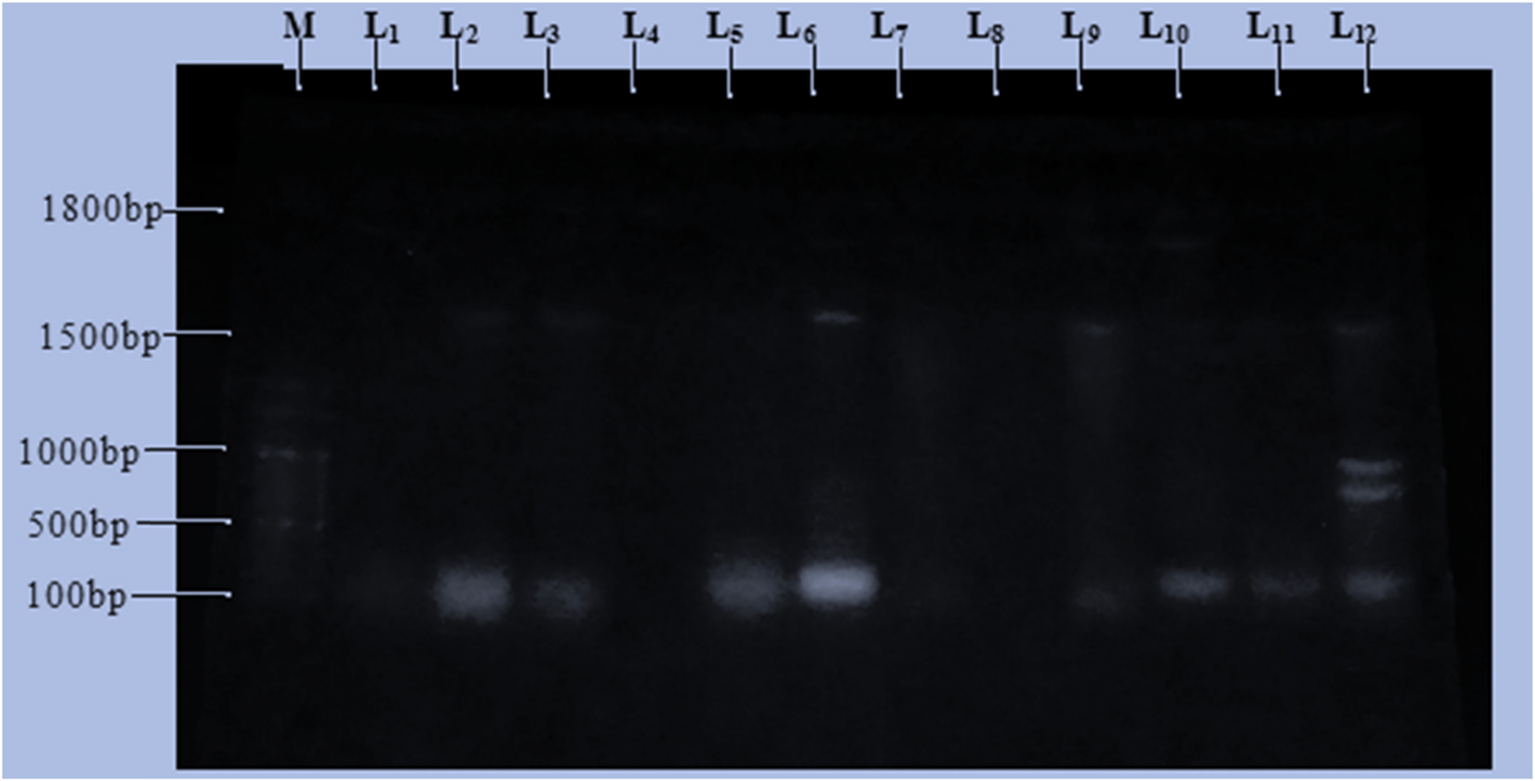
Plasmid size determination and a band size of 12 multi-drug-resistant bacteria. Key: M-Marker, L_1_-SD1, L_2_-SK5, L_3_-SD15, L_4_-SK8, L_5_-SK7, L_6_-SK3, L_7_-SD4, L_8_-SD7, L_9_-SD11, L_10_-SD2, L_11_-SK2, L_12_-SD16.

Plasmid isolation and profiling of the 12 bacterial isolates that were resistant to the commonly used broad-spectrum antibiotics were performed. Plasmid profiles for the bacterial isolates revealed that some possessed four plasmids, just a few had single plasmid, and two of them had no plasmid. Isolates SD1 (L_1_11), SK5 (L_2_), SD15 (L_3_), SK7(L_5_), SK3 (L_6_), SD11 (L_9_), SD2 (L_10_), SK2 (L_11_), and SD16 (L_12_) were having plasmid(s). Conversely, isolates SK8 (L_4_), SD4 (L_7_), and SD7 (L_8_) do not possess any plasmid when exposed to the horizontal gel electrophoresis, and it means that the multi-drug-resistant gene is not plasmid mediated but it may be chromosomal DNA-mediated. Therefore, the multi-drug resistance in these isolates may be plasmid or chromosomal DNA-mediated. The plasmid sizes for the isolates were found to have different base pair sizes (bps). SD1 (L_1_), SK7 (L_5_), and SK2 (L_11_) had one plasmid with a size of 100 bp, while SK5 (L_2_), SD15 (L_3_), SK3 (L_6_), and SD11 (L_9_) had two plasmid fragments of 100 bp and approximately 1,500 bp. SD16 (L_12_) had four plasmid sizes of 100, 900, 1,000, and approximately 1,500 bp, while SD2 (L_10_) contains two plasmids with sizes of 100 and approximately 1,800 bp. The result of the plasmid profile of the isolates is shown in Figure 3.

Plasmid profile studies are widely used for epidemiological studies, and the role of plasmids in drug resistance is well understood. In this study, the presence, number, and molecular size of plasmids were analyzed for the 12 multi-drug-resistant bacterial isolates. The isolates were found to contain various numbers of plasmids (1–4) with different sizes (from 100 to 1,800 bp) (Figure 1). The most common plasmid sizes encountered were 100 and 1,500 bps. These different-sized plasmids were responsible for the presence of multi-drug resistance. The isolates might acquire genetic elements (plasmids) from other bacteria. Large mobile genetic elements appear to encode both antibiotic-resistant factors and proteins that are responsible for the increase in virulence, thus giving the organism the ability to adapt to the selective pressure of antibiotics. This study correlates with the study done by Ojo et al. [21] and Olawale et al. [22]. The analysis of the plasmid profile makes it possible to estimate the dependence between antibiotic susceptibility and the presence of plasmids with respect to a particular isolate [20,22]. It can be said that resistance mediated by plasmid which has been observed in various studies such as Ojo et al. [21], Olawale et al. [22], Jaran [23], Agrahari et al. [24], Nmesirionye et al. [25], and Olorunfemi et al. [26] may be responsible for the differences observed in treatment by clinicians. The gene responsible for multi-drug resistance that occurred in bacterial isolates under this study may be grouped into chromosomal mediated [(SK8 (L_4_), SD4 (L_7_), and SD7 (L_8_)] and plasmid-mediated (the rest isolates).

### 3.5 Limitation of this study

The limitation of our study was the small number of sample sizes collected due to lack of resources.

## 4 Conclusions

This study aimed to investigate the presence of antibiotic resistance patterns and bacterial profiles of some multi-drug-resistant bacteria isolated from effluents of Kolladiba and Debark Hospitals. The findings reveal that effluents of Kolladiba and Debark Hospitals have a high incidence of multi-drug-resistant bacteria. From a total of 28 bacterial isolates, 12 were found to be multi-drug resistant. Among the tested antibiotics, erythromycin was the most resisted antibiotic, while novobiocin was the most effective antibiotic. A plasmid profile study of the isolates revealed both the presence and absence of plasmids. The number of plasmids ranged from zero to four, with plasmid sizes of 100, 900, 1,000, 1,400, 1,500, and 1,800 base pairs. Most of multi-drug resistances could be due to plasmid genes. To reduce the incidence of drug resistance, the unwise use of antibiotics by individuals whose health is impaired should be discouraged. In order to reduce the impact of hospital effluents on the spread of antimicrobial resistance in the community, a proper effluent treatment mechanism should be devised.
